# Are BMI and Sedentariness Correlated? A Multilevel Study in Children

**DOI:** 10.3390/nu7075258

**Published:** 2015-07-16

**Authors:** Thayse Natacha Gomes, Peter T. Katzmarzyk, Fernanda Karina dos Santos, Raquel Nichele de Chaves, Daniel Santos, Sara Pereira, Catherine M. Champagne, Donald Hedeker, José Maia

**Affiliations:** 1 Center of Research, Education, Innovation and Intervention in Sport (CIFI^2^D), Kinanthropometry Lab, Faculty of Sport, University of Porto, Rua Dr. Plácido Costa, 91, 4200-450 Porto, Portugal; E-Mails: thayse_natacha@hotmail.com (T.N.G.); d.monteiro.santos13@gmail.com (D.S.); sara.s.p@hotmail.com (S.P.); 2 Pennington Biomedical Research Center, Louisiana State University, 6400 Perkins Rd, Baton Rouge, LA 70808-4124, USA; E-Mails: Peter.Katzmarzyk@pbrc.edu (P.T.K.); catherine.champagne@pbrc.edu (C.M.C.); 3 Department of Physical Education and Sports Science, CAV, Federal University of Pernambuco (UFPE), Rua Alto do Reservatório, Bela Vista, 55608-680 Vitória de Santo Antão, PE, Brazil; E-Mail: fernandak.santos@hotmail.com; 4 Federal University of Technology—Paraná (UTFPR), Campus Curitiba, 80230-901 Curitiba, PR, Brazil; E-Mail: raquelnichele@live.com.pt; 5 Department of Public Health Sciences, University of Chicago Biological Sciences, 5841 S. Maryland Ave, Room W254, MC2000, Chicago, IL 60637, USA; E-Mail: DHedeker@health.bsd.uchicago.edu

**Keywords:** BMI, sedentariness, children, multilevel analysis

## Abstract

The purpose of this research was to investigate the relationship between body mass index (BMI) and sedentariness (Sed) in children and to examine the influence of child and school correlates on their variation. The sample comprises 580 children (337 girls, 9–11 years). Sedentariness was assessed with an accelerometer, and BMI was computed. Child- and school-level covariates were analyzed using multilevel models. No significant correlation between Sed and BMI was found. School context explains 5% and 1.5% of the total variance in Sed and BMI, respectively. At the child level, only moderate-to-vigorous physical activity was associated with both Sed (β = −0.02 ± 0.002) and BMI (β = −0.005 ± 0.002). Sleep time is related to Sed (β = −0.42 ± 0.04), while sex (β = 1.97 ± 0.13), biological maturity (β = 1.25 ± 0.07), media in the bedroom (β = 0.26 ± 0.08) and healthy (β = −0.09 ± 0.03) and unhealthy (β = −0.07 ± 0.04) diet scores were associated with BMI. None of the school-level covariates were related to BMI, but access to cafeteria (β = −0.97 ± 0.25), playground equipment (β = −0.67 ± 0.20) and restaurants (β = 0.16 ± 0.08) were related to Sed. In conclusion, Sed and BMI were not correlated. Further, they have different correlates, while children’s traits seem to play more relevant roles in their differences in Sed and BMI than the school milieu. This information should be taken into account when strategies to reduce Sed and BMI are implemented.

## 1. Introduction

Drastic increases in the prevalence of youth being overweight/obesity [1] and associated co-morbidities [2] have been reported in past decades. A systematic review [1] from 1980 to 2013 indicated that the prevalence of childhood obesity/children being overweight in developed countries increased from 16.2% to 22.6% and from 16.9% to 23.8% in boys and girls, respectively; in developing countries, this increase was from 8.1% to 12.9% in boys and from 8.4% to 13.4% in girls. Although still high, this increase appears to be stabilizing in some countries [3]. Since being overweight/obesity tends to track into adulthood [4], increasing risk for cardiovascular diseases and co-morbidities [5], excess weight in youth remains a major public health problem.

Sedentariness (Sed) is an emerging potential risk factor for obesity [6,7]. In a systematic review with the purpose of determining the relationship between sedentary behavior and health indicators in school-aged children, Tremblay *et al.* [7] reported that watching TV for more than 2 h per day was associated with unfavorable body composition; while in school-aged children, LeBlanc *et al*. [8] found low- to moderate-quality evidence to suggest that increased television viewing is associated with unfavorable levels of adiposity. Similarly, Prentice-Dunn and Prentice-Dunn [6], in a review, showed that, in general, sedentary behaviors were positively associated with weight status. Further compelling evidence suggests that youth spend large proportions of awake time in both screen-based sedentary behaviors (such as television viewing, using a computer, playing video games) and non-screen-based sedentary behaviors (such as educational sedentary behavior, motorized travel time, reading, playing a musical instrument, sitting, talking) [9] and that different kinds of sedentary behaviors have different correlates. In this context, Babey *et al.* [10] found that for time spent watching TV, variables positively correlated with its expression were male sex, ethnicity (namely American Indian and African American), lower household income, lower levels of physical activity, lower parental education and additional hours worked among parents; however, correlates of a greater amount of time spent using the computer were older age, ethnicity (Asian), higher household income, lower levels of physical activity, less parental knowledge of free-time activities and living in neighborhoods with higher proportions of non-white residents and higher proportions of low-income residents. In addition, it seems that there is no consensus regarding the correlates of sedentariness. On the one hand, Van der Horst [11], in a brief review, reported that, for adolescents, ethnicity (Caucasian), socioeconomic status and parent education were found to be inversely associated with adolescents’ sedentary behavior, but insufficient evidence was found to draw conclusions about its correlates for younger children. On the other hand, Uijtdewilligen *et al.* [12], also in a review, found insufficient evidence for determinants of sedentary behavior in both children and adolescents. Several interventions indicate that decreasing sedentary time can contribute to weight reduction in children [13,14,15], but results are not always similar. For example, a meta-analysis [13] found an overall significant effect size (g = −0.073; *p* = 0.021) of sedentary behavior intervention on BMI (taking into account interventions with multiple components (sedentary behavior, physical activity and diet) and interventions with a single component (sedentary behavior)), but the inclusion of physical activity promotion and/or diet improvement in the intervention program did not have a significant additive effect when compared to sedentary behavior alone. Furthermore, Epstein *et al.* [14] reported that interventions focused on decreasing sedentary behaviors or increasing physical activity were both associated with significant decreases in percent overweight and body fat. In addition, Tremblay *et al.* [7], conducting a meta-analysis for randomized controlled studies that aimed to reduce sedentary time and reported changes in BMI, showed a significant effect of −0.81 (95% CI of −1.44–−0.17, *p* = 0.01), indicating an overall decrease in mean BMI in response to the interventions.

BMI and Sed seemingly share common biological and environmental correlates [11,16,17], with sex [11,18], physical activity levels [19,20,21], maturity status [22], sleep time [23,24], family environment (namely siblings’ influence and availability of electronic media) [25,26,27], nutritional habits [28,29] and time spent at school [30,31] identified as possible determinants of Sed and/or BMI. Since children spend most of their awake time at school, school has a potential role in children’s Sed and BMI variation, offering ample opportunities for physical activity [32] and healthy eating [33].

The available evidence supports the hypothesis that Sed and BMI are correlated in youth. The present study aims to (1) study the relationship between BMI and Sed in Portuguese children and (2) investigate the importance of child and school correlates in BMI and Sed variation.

## 2. Methods

### 2.1. Sample

The present study sample is from the International Study of Childhood Obesity, Lifestyle and the Environment (ISCOLE), conducted in 12 countries from all major regions of the world, to determine the relationship between lifestyle behaviors and obesity and to investigate the influence of factors, including behavioral settings and physical, social and policy environments, on observed relationships within and between countries [34].

A total of 23 schools, from the North of Portugal, were enrolled in the ISCOLE project. In each school, all 5th grade students were invited to take part in the study, and those aged 9–11 years were considered eligible. From those, parental or legal guardian consent was obtained, and approximately 30–40 children (50% of each sex), per school, were randomly selected. The response rate was 95.7%.

School selection and inclusion in the project were completed systematically. Firstly, from a list provided by the North Regional Education Directory Board, eligible public schools were selected by location in different regions and socio-economic neighborhoods. Secondly, selected schools were contacted, the project presented to the Physical Education Department coordinator, to the Physical Education Department, to the school Principal and Pedagogical Council and the Parental Council and approval obtained prior to implementation. Thirdly, parental or legal guardian consents were sent to all 5th grade students for signature. The study protocol was approved by the University of Porto ethics committee and by each school’s directorate councils (Physical Education Department, school Principal and Parental Council).

### 2.2. Outcome Variables

Our first outcome variable was Sed, objectively measured using ActiGraph GT3X+ accelerometers (ActiGraph, Pensacola, FL, USA). Children were instructed to wear the accelerometer at their waist on an elasticized belt placed on the right mid-axillary line 24 h day^−1^, for at least 7 days (including during sleep), removing the device only when performing activities in contact with water (*i.e.*, taking a shower, swimming). For eligibility, children were required to have at least 4 days (from which at least one was a weekend day) with a minimum of 10 h of daily awake wear time. Accelerometer information was divided into daytime activities and nocturnal sleep time using a validated algorithm [35,36]. Non-wear time during awake periods was defined as any sequence of at least 20 consecutive minutes of zero activity counts [36]. Mean week sedentary time (min·day^−1^) was defined as ≤25 activity counts per 15-s epoch [37].

Our second outcome variable was BMI. Height and weight were measured according to standardized ISCOLE procedures and instrumentation [34]. For height and sitting height, children were without shoes, with heads positioned to the Frankfurt plane, using a Seca 213 portable stadiometer rounding up to the nearest 0.1 cm (Seca, Hamburg, Germany). For height, children were fully erect, feet together, and the measurement was taken at the end of a deep inhalation, while for sitting height, children were seated on a table with legs hanging freely and arms resting on the thighs. Body weight was measured using a portable Tanita SC-240 Body Composition Analyzer scale (Tanita^®^, Arlington Heights, IL, USA), on children without shoes and socks and wearing light clothes. Measurements were taken twice and the average used for analysis. A third measurement was obtained if the difference between the previous two measurements was outside the permissible range for each measurement and its replica (0.5 cm for height and sitting height; 0.5 kg for weight), and in this case, the closest two measurements were averaged and used for analysis. BMI was computed using the standardized formula (weight (kg)/height (m^2^)).

### 2.3. Predictor Variables

#### 2.3.1. At the Child Level

Predictor variables at the child level included sex, biological maturation, moderate-to-vigorous physical activity (MVPA), sleep time, dietary patterns and family demographics. Biological maturation was computed using maturity offset sex-specific regression equations [38]. This method estimates the timing to peak height velocity (PHV) occurrence in decimal years. A positive (+) maturity offset is the number of years a child is beyond PHV; while a negative (−) maturity offset is the number of years a child is before the PHV; a zero value indicates that a child is experiencing his/her PHV. Although using the maturity offset estimates it is possible to classify children in their maturity status based on estimated age of PHV, *i.e.*, delayed, on time and advanced, we chose not to do so, given the narrow age range of the sample: 9–11.

Weekly mean MVPA (min·day^−1^) and sleep time (h·day^−1^) were estimated by accelerometry. MVPA was defined as activities greater or equal to 574 activity counts per 15-s epoch [37]; nocturnal sleep time for participants was determined using a novel and fully-automated algorithm specifically developed for use in ISCOLE and epidemiological studies employing 24-h waist-worn accelerometry [35,36].

Information on dietary patterns was obtained from questionnaires [34], completed on the same day as anthropometric measures. The questionnaire included frequency of consumption of different foods in a typical week, specifically fruits, vegetables, sweets, soft drinks, other foods and fast food consumption. Reported frequencies were converted into portions/week for principal components analysis. The component scores computed for each subject for two dietary patterns were standardized to ensure normality. These two patterns were designated as “unhealthy” (positive loadings for hamburgers, soft drink, fried food, *etc.*) and “healthy” (positive loadings for vegetables, fruits, *etc.*).

Basic family demographics were obtained via questionnaire, completed by parents or legal guardians (SCOLE Demographic and Family Health Questionnaire [34]), for information on ethnicity, family health and socioeconomic factors. Media availability in the child’s bedroom included computer or video games or a TV in their bedroom. A “media bedroom” variable classified the child as “having media” or “not having media” in the bedroom. Family size, including other siblings, was also used.

#### 2.3.2. School Level

The ISCOLE School Environment Questionnaire [34] was completed by the physical education teacher or school principal. Environmental aspects of the school were considered: students’ access to (i) outdoor facilities outside of school hours; (ii) playground equipment during school hours; (iii) cafeteria at school; (iv) fast food restaurants close to schools and (v) drink vending machines. In addition, information regarding the daily time each child spends at school was used, and a “mean week school time” was calculated.

Data were collected from September 2011 to January 2013, and all assessments were completed with a single week at each school by trained personnel from the Kinanthropometry Laboratory of the Faculty of Sport (University of Porto) following certification by the ISCOLE Coordinating Center. After the inclusion criteria (children with accelerometer valid data for ≥4 days and with no missing information in any other variables), the final sample included 580 children (337 girls).

### 2.4. Data Analysis

For exploratory and descriptive statistics, SPSS 21 was used. To address our first aim, a multivariate multilevel model, as suggested by Goldstein [39] and Snijders and Bosker [40], for situations where at least two outcome variables are used at the same time (in our case Sed and BMI), was employed. These and others [41,42] have outlined the main reasons for such an approach, specifically increased power, reduction in capitalization by chance with systematic testing and ability to model correlations (at school and child levels) between outcome variables.

If the correlations at the child and school level in our joint outcomes were observed to be not statistically significant, then a univariate outcome analysis (BMI separated from Sed) using child and school covariates within a multilevel approach was conducted. A three-step analytical approach was used [40,41]. In Step 1 (our null model), only an intercept term and variances (at school and child levels) were estimated. These variance estimates allowed us to calculate the total variation in Sed and in BMI explained at the school level. In Step 2 (Model 1), child-level predictors were included, and in Step 3 (Model 2), school context variables were added. Final decisions were made according to deviance and corresponding likelihood-ratio (LR) tests in nested models of increasing complexity. A more complex model fits better than the previous one if the difference in their respective deviances is statistically significant by the LR test, according to a chi-square statistic with degrees of freedom equal to the difference in estimated parameters between the two models. Since BMI is kg·m^−2^ and Sed is min·day^−1^, to make all fixed effects coefficients, as well as variances, comparable for BMI with Sed, we standardized BMI and Sed, *i.e.*, expressed in z-scores. All multilevel (multivariate and univariate) analyses were done in SuperMix software v.1 [43], and for the ease of interpretation, all predictors were centered on the grand mean. Explicit formulation of these types of models and estimation details are described elsewhere [39,40].

## 3. Results

Descriptive statistics (mean ± SD and percentage) are presented in Table 1. On average, children spent about 9.5 h·day^−1^ or about 1/3 of the day in sedentary activities, <1 h·day^−1^ in MVPA, and slept about 8 h·night^−1^. They were about two years from PHV, and spent ≈7 h·day^−1^ at school. In terms of overweight/obesity prevalence, 42.4% of girls and 47.3% of boys are overweight/obese, with the average of the student population at 44.5%. Significant differences among sexes were observed for sedentary time, MVPA, maturation, sleep and dietary pattern scores. Compared to boys, girls are ahead in their maturity status, spend more time in sedentary activities and sleep and less time on MVPA, have higher healthy diet scores and lower unhealthy diet scores. Children had about one sibling on average; almost 81% had media in their bedroom.

Almost 96% of schools reported that students had access to a cafeteria, and 69.6% reported that students had access to fast food restaurants close to school. However, only 26.1% reported that students had access to vending machines. About half of the schools allowed students access to sports equipment outside school hours; only 8.7% of them allowed students access to playground equipment during school hours.

On average, the children in this sample spent 560 ± 4 min·day^−1^ in sedentary behavior and had a BMI of 19.3 ± 0.2 kg·m^−2^ (Table 2). Schools explain small amounts of the total variation of Sed (4.9%) and BMI (1.5%). The major portion of the total variance in Sed and BMI is at the child level. Interestingly, at the child level, the covariance between Sed and BMI is not significant (σ_SED, BMI_ = −1.91 ± 0.83, *p* = 0.826), translating to a small and negative correlation coefficient (ρ_SED, BMI_ = −0.01).

**Table 1:** Descriptive statistics for variables at the child (level 1) and school level (level 2).

Child-level Variables (mean ± SD or percentage)				
		Boys	Girls	Total
BMI (kg·m^−2^)		19.2 ± 3.3	19.3 ± 3.4	19.3 ± 3.4
Sedentary mean (min·day^−1^)		544 ± 66 *	572 ± 59	560 ± 63
MVPA (min·day^−1^)		67 ± 23 *	46 ± 15	55 ± 22
Maturity Offset (years to PHV)		−2.78 ± 0.42 *	−1.25 ± 0.53	−1.89 ± 0.90
Sleep time (h·day^−1^)		8.2 ± 0.9 *	8.4 ± 0.8	8.3 ± 0.9
Number of siblings		0.98 ± 0.80	0.96 ± 0.82	0.96 ± 0.82
Healthy diet score		−0.09 ± 1.00 *	0.11 ± 0.99	0.03 ± 1.00
Unhealthy diet score		0.21 ± 1.21 *	−0.21 ± 0.68	−0.04 ± 0.96
Time at school (h·day^−1^)		6.8 ± 0.4	6.8 ± 0.4	6.8 ± 0.4
BMI Classification				
Normal-weight		57.6%	42.4%	55.5%
Overweight/obese		52.7%	47.3%	44.5%
Media in Bedroom				
No		18.1%	19.9%	19.1%
Yes		81.9%	80.1%	80.9%
**School-level Variables (percentage)**				
Student’s access to cafeteria				
No				4.3%
Yes				95.7%
Student’s access to fast food restaurant				
No				30.4%
Yes				69.6%
Student’s access to drink vending machines				
No				73.9%
Yes				26.1%
Student’s access to playground equipment during school hours				
No				91.3%
Yes				8.7%
Student’s access to sports equipment outside school hours				
No				47.8%
Yes				52.2%

* *p* < 0.05. MVPA, moderate-to-vigorous physical activity; PHV, peak height velocity.

**Table 2:** Null model main results (parameter estimates, standard errors (SE) and deviance) for both sedentariness (Sed) and BMI.

	Sed		BMI		Covariance	
	Estimate	SE	Estimate	SE	Estimate	SE
Fixed Effects						
Intercept	559.68 *	3.93	19.26 *	0.16		
Random Effects						
School-level						
Variance	196.81 *	104.24	0.17 ^ns^	0.18		
Covariance (σ_SL_)					1.68 ^ns^	3.07
Correlation (ρ_SL_)					0.29 ^ns^	
Child-level						
Variance	3818.05 *	228.71	11.03 *	0.66		
Covariance (σ_CL_)					−1.91 ^ns^	0.83
Correlation (ρ_CL_)					−0.01 ^ns^	
Deviance		9493.6327				

* *p* < 0.05; ns = not significant. School explained variance for Sed = [196.807/(196.807 + 3818.052)] = 4.9%; and for BMI = [0.166/(0.166 + 11.029)] = 1.5%.

Since no statistically-significant covariance/correlation was observed between Sed and BMI at the child level, a separate multilevel analysis for each outcome variable using values transformed into *z*-scores was computed. Predictor variables from both child and school levels were used.

The null model 0028 (Table 3) indicated that school-level effects, expressed as intraclass correlation coefficients, were 0.050 and 0.015 for Sed and BMI, respectively; 5% of variance in Sed and 1.5% of variance in BMI among children are explained by school effects; 95% of Sed variance and 98.5% of BMI variance are explained by child-level characteristics.

Results from Model 1 (Table 3) indicate that sleep time was significantly associated with Sed: children who sleep more (β = −0.43, SE = 0.04, *p* < 0.001) were less sedentary; no significant effect of sleep time on BMI was observed. Sex, biological maturity, bedroom media availability and dietary patterns correlated with BMI, but not Sed: boys (β = 1.97, SE = 0.13, *p* < 0.001), children advanced in biological maturity (β = 1.25, SE = 0.07, *p* < 0.001) and those with media in the bedroom (β = 0.26, SE = 0.08, *p* = 0.002) had higher BMI than girls, than later maturing children and those without media in the bedroom, respectively. Children with a higher healthy diet score (β = −0.09, SE = 0.03, *p* = 0.009) tended to have lower BMI, also true for the unhealthy diet score, but marginally significant (β = −0.07, SE = 0.03, *p* = 0.054). MVPA was the only variable significantly correlated with both Sed and BMI, where higher MVPA involvement was negatively related to both (for Sed: β = −0.02, SE = 0.002, *p* < 0.001; for BMI: β = −0.004, SE = 0.002, *p* = 0.011); the effect of MVPA is greater in the reduction of Sed compared to BMI.

**Table 3:** Results summary of modelling Sed and BMI: estimates ^¥^ (standard errors).

	Sed			BMI		
Parameters	Null Model	Model 1	Model 2	Null Model	Model 1	Model 2
	Fixed Effects					
Intercept	−0.008 ^§^ (0.062)	0.083 (0.14)	1.05 (0.28) *	−0.004 ^‡^ (0.05)	1.34 (0.13) *	1.11 (0.27) *
Sex		0.06 (0.13)	0.05 (0.13)		1.97 (0.13) *	1.97 (0.13) *
Siblings		−0.03 (0.04)	−0.02 (0.04)		−0.01 (0.04)	−0.02 (0.04)
Maturity offset		0.07 (0.07)	0.07 (0.07)		1.25 (0.07) *	1.25 (0.07) *
Media bedroom		0.01 (0.09)	0.01 (0.09)		0.26 (0.08) *	0.26 (0.08) *
MVPA		−0.02 (0.002) *	−0.02 (0.002) *		−0.004 (0.002) *	−0.005 (0.002) *
Sleep time		−0.43 (0.04) *	−0.42 (0.04) *		−0.02 (0.04)	−0.02 (0.04)
Healthy diet score		0.02 (0.03)	0.03 (0.03)		−0.09 (0.03) *	−0.09 (0.03) *
Unhealthy diet score		−0.03 (0.04)	−0.03 (0.04)		−0.07 (0.04) **	−0.07 (0.04) *
Time at school		−0.06 (0.1)	−0.08 (0.09)		0.13 (0.08) **	0.09 (0.09)
Access to cafeteria			−0.97 (0.25) *			0.24 (0.24)
Access to fast food restaurant			0.16 (0.08) *			0.04 (0.08)
Access to drink vending machine			−0.11 (0.09)			0.02 (0.09)
Access to playground equipment			−0.67 (0.20) *			0.08 (0.19)
Access to sport equipment outside school hour			−0.12 (0.08)			−0.06 (0.08)
	Random Effects					
Between-school variance	0.05 (0.03)	0.03 (0.02)	0.007 (0.007)	0.02 (0.02)	0.003 (0.007)	0.003 (0.007)
Within-school (child) variance	0.95 (0.06)	0.63 (0.04)	0.63 (0.04)	0.98 (0.06)	0.60 (0.04)	0.59 (0.04)
	Model Summary					
Deviance	1634.4114	1396.9434	1374.8328	1643.2667	1342.2034	1340.4157
Number of estimated parameters	3	12	17	3	12	17

^¥^ All estimates are expressed as z-scores; ^§^ =559.68 min∙day^−1^ in the original metric; ^‡^ =19.26 kg∙m^−2^ in the original metric; * *p* < 0.05; ** *p* < 0.10.

The difference in deviance from the null model to Model 1 (for sedentariness: Δ = 237.468, nine degrees of freedom; for BMI: Δ = 301.0633, nine degrees of freedom) was statistically significant; therefore, Model 1 fits better than the null model in explaining the variance of each outcome variable. Further, from Model 1, the proportion of variance in Sed and BMI explained by children’s characteristics was 48% for Sed and 80% for BMI.

Children with access to a cafeteria (β = −0.97, SE = 0.25, *p* < 0.001) and those with access to playground equipment (β = −0.67, SE = 0.20, *p* < 0.001) were less sedentary; children with access to fast food restaurants close to school (β = 0.16, SE = 0.08, *p* = 0.037) were more sedentary. However, none of the school-level covariates were related to BMI.

The difference in deviance from Model 1 to Model 2 (for Sed: Δ = 22.1015, five degrees of freedom; for BMI: Δ = 1.7866, five degrees of freedom), was only significant for Sed. For Sed, Model 2 fits better than Model 1; but for BMI, Model 1 is the best. Approximately 86% of the original 5% of the between-school variance in Sed was attributed to students having access to cafeteria, playground equipment during school hours and fast food restaurants close to school.

## 4. Discussion

The relationship between BMI and Sed in a sample of Portuguese children and child- and school-level correlates using both multivariate and univariate multilevel models was explored. Results from the multivariate model indicated that correlations between BMI and Sed were low and not statistically significant. Relationships between Sed and BMI reported in youth suggest that greater time spent in Sed behavior (especially time watching TV) is associated with higher body weight [7]. However, this relationship was not found, and that may be related to the fact that mean total Sed time was used in the analysis, rather than specific sedentary behaviors (such as watching TV, using the computer, playing video games, doing homework). Carandente *et al*. [44] found positive correlations between BMI and time spent in sedentary activities, and between time spent in sedentariness and food consumption; the more hours 8–10-year-old children spent watching TV, the more likely they consumed snacks and beverages. Watching TV and other sedentary behaviors may stimulate eating and increased energy intake, thus affecting body weight [15]. Results suggest that the relationship between body weight and Sed may be indirect, with eating behaviors and energy intake mediating this relationship.

Our univariate multilevel analysis indicated that from all of the child-level predictors, only MVPA was significantly associated with both Sed and BMI. Previous research offered similar results regarding BMI; at least 1 h of daily MVPA was shown to reduce the likelihood of being overweight/obesity in Portuguese children [19]. Others [20] found that increased MVPA reduced BMI z-scores over three years in overweight/obese children. In examining the relationships between being overweight, diet and physical activity patterns in youth, Janssen *et al*. [45] concluded that increasing involvement in physical activity is a relevant strategy to prevent/treat excess weight. Previous studies have reported similar MVPA and Sed results [21], and as Epstein and Roemmich [46] note, engagement in physical activity “usually involves choosing exercise over a concurrent and powerful competing sedentary behavior” (p. 103). Since we found stronger effects of MVPA in reducing Sed, than BMI, other factors beyond behavioral, namely genetic, have key roles in increasing/decreasing BMI [47].

Sleep time is relevant to children’s health [23,24,48,49]. However, associations between sleep and Sed are inconclusive [23,49]. No relationship was found in Taiwanese adolescents [49] between time spent watching TV/using the computer and sleep; however, among Belgium students [23], those spending more time in sedentary activities spent less time in bed on weekdays. In our data, the negative association between sleep and Sed suggests that since hours of the day are limited, sleeping more reduces available sedentary time [50]. Although there is evidence of an association between short sleep duration and obesity in youth [24,48], this was not observed in our study.

Other predictors at the child level (except number of siblings and time spent at school), sex, biological maturation, bedroom media availability and diet, were only associated with BMI, similar to previous reports. Among Spanish youth [51], a significantly higher prevalence of obesity was observed for boys. Furthermore, the relationship between BMI and maturity status seems to be clear: more mature children tend to be taller and heavier [22].

Screen time is frequently researched in children, usually negatively associated with BMI [25]; and having a TV in the bedroom increases the risk of being overweight/obese [26,27]. Our results reinforce this: on average, children with bedroom media had higher BMI than those without. While unclear, several mechanisms possibly contribute: reduced energy expenditure while watching TV, increased dietary intake through snacking and increased exposure to media promoting food consumption. Higher healthy diet pattern scores were negatively associated with BMI. Differences in diet are not always observed between normal and overweight children [28,29]. However, normal weight children may consume significantly more carbohydrate and fiber and less fat and high calorie beverages compared to overweight peers.

The school environment, widely recognized as promoting active and health lifestyles among children/adolescents, offers mandatory/extracurricular activities and policies reducing sedentary time [32]. In the present study, 5% of variance in Sed was explained by the school environment. Playground areas [32] provide opportunities to engage in physical activity during recess and reduce sedentary time. Promoting healthy eating and access to food at school affect weight gain and control [33]. However, no relationship between students’ access to cafeteria or fast food close to school and BMI was found, but a significant association between these two predictors and Sed was observed.

School “effects” on children’s BMI was only 1.5%. Pallan *et al*. [52] similarly found low intraclass correlations (*i.e.*, variance attributable to school effects), varying from 0.9% to 4.2%. Low school-level variation in Sed has previously been reported [53]. Relatively, low numbers of schools (23) and low variance across Portuguese school environments may explain the low school effects on Sed and very low results on BMI.

There are study limitations: (1) its cross-sectional nature does not allow cause-and-effect interpretations; (2) the number of schools and low variance of schools’ contextual characteristics limit identification of school-level traits on BMI and Sed; (3) no information on the home or neighborhood environment as child-level predictors was considered; (4) one Portuguese regional population limits generalization, although overweight/obesity prevalence [54] and socioeconomic status distribution [55] compare with previous studies; (5) diet as a mediated variable in the relationship between Sed and BMI in multilevel models was not used; and (6) since all children had at least 10 h of awake wear time, with a mean accelerometer use value of 15.17 ± 0.86 h, we did not adjust physical activity and Sed for wear time, because this effect is not significant (data not shown). The strengths were: (1) multivariate multilevel analysis identifying relationships between Sed and BMI, with multilevel modelling to understand complex nested information at child and school levels; (2) objective methods to estimate Sed, MVPA and sleep time; (3) standardized data collection methods; and (4) reliable child- and school-level information.

## 5. Conclusions

Sed and BMI were not significantly correlated, but MVPA is significantly associated with both. However, correlations were different and should be considered, since strategies to reduce Sed or BMI may act through different pathways. Low variance at the school level for both BMI and Sed reinforce suggestions that although children spend considerable awake time at school, individual variables play more relevant roles in differences between Sed and BMI than school. School policies promoting active and healthy habits play important roles in reducing sedentary time, making wise nutritional choices and controlling body weight.

## References

[1] Ng M., Fleming T., Robinson M., Thomson B., Graetz N., Margono C., Mullany E.C., Biryukov S., Abbafati C., Abera S.F. (2014). Global, regional, and national prevalence of overweight and obesity in children and adults during 1980–2013: A systematic analysis for the global burden of disease study 2013. Lancet.

[2] Huang T.T., Ball G.D., Franks P.W. (2007). Metabolic syndrome in youth: Current issues and challenges. Appl. Physiol. Nutr. Metab..

[3] Olds T., Maher C., Zumin S., Peneau S., Lioret S., Castetbon K., Bellisle, de Wilde J., Hohepa M., Maddison R. (2011). Evidence that the prevalence of childhood overweight is plateauing: Data from nine countries. Int. J. Pediatr. Obes..

[4] Deshmukh-Taskar P., Nicklas T.A., Morales M., Yang S.J., Zakeri I., Berenson G.S. (2006). Tracking of overweight status from childhood to young adulthood: The bogalusa heart study. Eur. J. Clin. Nutr..

[5] Berenson G.S., Srnivasan S.R. (2005). Cardiovascular risk factors in youth with implications for aging: The bogalusa heart study. Neurobiol. Aging.

[6] Prentice-Dunn H., Prentice-Dunn S. (2012). Physical activity, sedentary behavior, and childhood obesity: A review of cross-sectional studies. Psychol. Health Med..

[7] Tremblay M.S., LeBlanc A.G., Kho M.E., Saunders T.J., Larouche R., Colley R.C., Goldfield G., Connor Gorber S. (2011). Systematic review of sedentary behaviour and health indicators in school-aged children and youth. Int. J. Behav. Nutr. Phys. Act..

[8] LeBlanc A.G., Spence J.C., Carson V., Connor Gorber S., Dillman C., Janssen I., Kho M.E., Stearns J.A., Timmons B.W., Tremblay M.S. (2012). Systematic review of sedentary behaviour and health indicators in the early years (aged 0–4 years). Appl. Physiol. Nutr. Metab..

[9] Pate R.R., Mitchell J.A., Byun W., Dowda M. (2011). Sedentary behaviour in youth. Br. J. Sports Med..

[10] Babey S.H., Hastert T.A., Wolstein J. (2013). Adolescent sedentary behaviors: Correlates differ for television viewing and computer use. J. Adolesc. Health.

[11] Van Der Horst K., Paw M.J., Twisk J.W., Van Mechelen W. (2007). A brief review on correlates of physical activity and sedentariness in youth. Med. Sci. Sports Exerc..

[12] Uijtdewilligen L., Nauta J., Singh A.S., van Mechelen W., Twisk J.W., van der Horst K., Chinapaw M.J. (2011). Determinants of physical activity and sedentary behaviour in young people: A review and quality synthesis of prospective studies. Br. J. Sports Med..

[13] Liao Y., Liao J., Durand C.P., Dunton G.F. (2014). Which type of sedentary behaviour intervention is more effective at reducing body mass index in children? A meta-analytic review. Obes. Rev..

[14] Epstein L.H., Paluch R.A., Gordy C.C., Dorn J. (2000). Decreasing sedentary behaviors in treating pediatric obesity. Arch. Pediatr. Adolesc. Med..

[15] Epstein L.H., Valoski A.M., Vara L.S., McCurley J., Wisniewski L., Kalarchian M.A., Klein K.R., Shrager L.R. (1995). Effects of decreasing sedentary behavior and increasing activity on weight change in obese children. Health Psychol..

[16] Gupta N., Goel K., Shah P., Misra A. (2012). Childhood obesity in developing countries: Epidemiology, determinants, and prevention. Endocr. Rev..

[17] Dunton G.F., Kaplan J., Wolch J., Jerrett M., Reynolds K.D. (2009). Physical environmental correlates of childhood obesity: A systematic review. Obes. Rev..

[18] Meigen C., Keller A., Gausche R., Kromeyer-Hauschild K., Bluher S., Kiess W., Keller E. (2008). Secular trends in body mass index in german children and adolescents: A cross-sectional data analysis via crescnet between 1999 and 2006. Metabolism.

[19] Bingham D.D., Varela-Silva M.I., Ferrao M.M., Augusta G., Mourao M.I., Nogueira H., Marques V.R., Padez C. (2013). Socio-demographic and behavioral risk factors associated with the high prevalence of overweight and obesity in portuguese children. Am. J. Hum. Biol..

[20] Trinh A., Campbell M., Ukoumunne O.C., Gerner B., Wake M. (2013). Physical activity and 3-year bmi change in overweight and obese children. Pediatrics.

[21] Raudsepp L., Neissaar I., Kull M. (2008). Longitudinal stability of sedentary behaviors and physical activity during early adolescence. Pediatr. Exerc. Sci..

[22] Malina R.M., Bouchard C., Bar-Or O. (2004). Growth, Maturation and Physical Activiy.

[23] Van den Bulck J. (2004). Television viewing, computer game playing, and internet use and self-reported time to bed and time out of bed in secondary-school children. Sleep.

[24] Patel S.R., Hu F.B. (2008). Short sleep duration and weight gain: A systematic review. Obesity (Silver Spring).

[25] Must A., Tybor D.J. (2005). Physical activity and sedentary behavior: A review of longitudinal studies of weight and adiposity in youth. Int. J. Obes. (Lond.).

[26] Adachi-Mejia A.M., Longacre M.R., Gibson J.J., Beach M.L., Titus-Ernstoff L.T., Dalton M.A. (2007). Children with a TV in their bedroom at higher risk for being overweight. Int. J. Obes..

[27] Delmas C., Platat C., Schweitzer B., Wagner A., Oujaa M., Simon C. (2007). Association between television in bedroom and adiposity throughout adolescence. Obesity (Silver Spring).

[28] Yannakoulia M., Brussee S.E., Drichoutis A.C., Kalea A.Z., Yiannakouris N., Matalas A.L., Klimis-Zacas D. (2012). Food consumption patterns in mediterranean adolescents: Are there differences between overweight and normal-weight adolescents?. J. Nutr. Educ. Behav..

[29] Maier I.B., Ozel Y., Wagnerberger S., Bischoff S.C., Bergheim I. (2013). Dietary pattern and leisure time activity of overweight and normal weight children in germany: Sex-specific differences. Nutr. J..

[30] Harrington D.M., Dowd K.P., Bourke A.K., Donnelly A.E. (2011). Cross-sectional analysis of levels and patterns of objectively measured sedentary time in adolescent females. Int. J. Behav. Nutr. Phys. Act..

[31] Steele R.M., van Sluijs E.M., Sharp S.J., Landsbaugh J.R., Ekelund U., Griffin S.J. (2010). An investigation of patterns of children’s sedentary and vigorous physical activity throughout the week. Int. J. Behav. Nutr. Phys. Act..

[32] Ridgers N.D., Stratton G., Fairclough S.J., Twisk J.W. (2007). Long-term effects of a playground markings and physical structures on children’s recess physical activity levels. Prev. Med..

[33] Wechsler H., Devereaux R.S., Davis M., Collins J. (2000). Using the school environment to promote physical activity and healthy eating. Prev. Med..

[34] Katzmarzyk P.T., Barreira T.V., Broyles S.T., Champagne C.M., Chaput J.P., Fogelholm M., Hu G., Johnson W.D., Kuriyan R., Kurpad A. (2013). The international study of childhood obesity, lifestyle and the environment (iscole): Design and methods. BMC Public Health.

[35] Tudor-Locke C., Barreira T.V., Schuna J.M., Mire E.F., Katzmarzyk P.T. (2014). Fully automated waist-worn accelerometer algorithm for detecting children’s sleep-period time separate from 24-h physical activity or sedentary behaviors. Appl. Physiol. Nutr. Metab..

[36] Barreira T.V., Schuna J.M., Mire E.F., Katzmarzyk P.T., Chaput J.-P., Leduc G., Tudor-Locke C. (2015). Identifying children’s nocturnal sleep using 24-h waist accelerometry. Med. Sci. Sports Exerc..

[37] Evenson K.R., Catellier D.J., Gill K., Ondrak K.S., McMurray R.G. (2008). Calibration of two objective measures of physical activity for children. J. Sports Sci..

[38] Mirwald R.L., Baxter-Jones A.D., Bailey D.A., Beunen G.P. (2002). An assessment of maturity from anthropometric measurements. Med. Sci. Sports Exerc..

[39] Goldstein H. (2003). Multilevel Statistical Models.

[40] Snijders T., Bosker R. (2012). Multilevel Analysis. An Introduction to Basic and Advanced Multilevel Modeling.

[41] Hox J. (2010). Multilevel Analysis. Techniques and Applications.

[42] Tabachnick B., Fidell L. (2007). Using Multivariate Statistics.

[43] Hedeker D., Gibbons R., du Toit M., Cheng Y. (2008). Supermix for Mixed Effects Models.

[44] Carandente F., Roveda E., Montaruli A., Pizzini G. (2009). Nutrition, activity behavior and body constitution in primary school children. Biol. Sport.

[45] Janssen I., Katzmarzyk P.T., Boyce W.F., Vereecken C., Mulvihill C., Roberts C., Currie C., Pickett W. (2005). Comparison of overweight and obesity prevalence in school-aged youth from 34 countries and their relationships with physical activity and dietary patterns. Obes. Rev..

[46] Epstein L.H., Roemmich J.N. (2001). Reducing sedentary behavior: Role in modifying physical activity. Exerc. Sport Sci. Rev..

[47] Butte N.F., Cai G., Cole S.A., Comuzzie A.G. (2006). Viva la familia study: Genetic and environmental contributions to childhood obesity and its comorbidities in the hispanic population. Am. J. Clin. Nutr..

[48] Knutson K.L., Spiegel K., Penev P., Van Cauter E. (2007). The metabolic consequences of sleep deprivation. Sleep Med. Rev..

[49] Chen M.Y., Wang E.K., Jeng Y.J. (2006). Adequate sleep among adolescents is positively associated with health status and health-related behaviors. BMC Public Health.

[50] Olds T., Ferrar K.E., Gomersall S.R., Maher C., Walters J.L. (2012). The elasticity of time: Associations between physical activity and use of time in adolescents. Health Educ. Behav..

[51] Serra-Majem L., Aranceta Bartrina J., Perez-Rodrigo C., Ribas-Barba L., Delgado-Rubio A. (2006). Prevalence and deteminants of obesity in spanish children and young people. Br. J. Nutr..

[52] Pallan M.J., Adab P., Sitch A.J., Aveyard P. (2014). Are school physical activity characteristics associated with weight status in primary school children? A multilevel cross-sectional analysis of routine surveillance data. Arch. Dis. Child..

[53] Gomes T.N., dos Santos F.K., Santos D., Pereira S., Chaves R., Katzmarzyk P.T., Maia J. (2014). Correlates of sedentary time in children: A multilevel modelling approach. BMC Public Health.

[54] Sardinha L.B., Santos R., Vale S., Silva A.M., Ferreira J.P., Raimundo A.M., Moreira H., Baptista F., Mota J. (2011). Prevalence of overweight and obesity among portuguese youth: A study in a representative sample of 10–18-year-old children and adolescents. Int. J. Pediatr. Obes..

[55] Fundação Francisco Sá Carneiro (2013). Fundação Francisco Manuel dos Santos. Pordata. www.pordata.pt.

